# The Diseases of Live Stock and Their Most Efficient Remedies

**Published:** 1879-04

**Authors:** 


THE DISEASES OF LIVE STOCK AND THEIR MOST EFFICIENT REMEDIES, Including horses, cattle, sheep and swine. Being a popular treatise, giving in brief and plain language a description of all the usual diseases to which these animals are liable, and the most successful treatment by American, English and European veterinarians. By LLOYD V. TELLOR, M.D. D. G. Brinton, 115 S. Seventh street, Philadelphia. 1879.

A first class hand book of veterinary practice, with every disease that animals are subject to arranged in systematic order and treated of in a plain, comprehensive manner. All who deal in live stock should have the work. It is adapted particularly to the use of farmers, etc., and all technicalities are avoided.

